# A staphylococcal anti-sigma factor possesses a single-domain, carries different denaturant-sensitive regions and unfolds via two intermediates

**DOI:** 10.1371/journal.pone.0195416

**Published:** 2018-04-05

**Authors:** Debabrata Sinha, Rajkrishna Mondal, Avisek Mahapa, Keya Sau, Rajagopal Chattopadhyaya, Subrata Sau

**Affiliations:** 1 Department of Biochemistry, Bose Institute, Kolkata, West Bengal, India; 2 Department of Biotechnology, Nagaland University, Dimapur, Nagaland, India; 3 Department of Biotechnology, Haldia Institute of Technology, Haldia, West Bengal, India; Russian Academy of Medical Sciences, RUSSIAN FEDERATION

## Abstract

RsbW, an anti-sigma factor possessing kinase activity, is expressed by many Gram-positive bacteria including *Staphylococcus aureus*. To obtain clues about the domain structure and the folding-unfolding mechanism of RsbW, we have elaborately studied rRsbW, a recombinant *S*. *aureus* RsbW. Sequence analysis of the protein fragments, generated by the limited proteolysis of rRsbW, has proposed it to be a single-domain protein. The unfolding of rRsbW in the presence of GdnCl or urea was completely reversible in nature and occurred through the formation of at least two intermediates. The structure, shape, and the surface hydrophobicity of no intermediate completely matches with those of other intermediates or the native rRsbW. Interestingly, one of the intermediates, formed in the presence of less GdnCl concentrations, has a molten globule-like structure. Conversely, all of the intermediates, like native rRsbW, exist as dimers in aqueous solution. The putative molten globule and the urea-generated intermediates also have retained some kinase activity. Additionally, the putative ATP binding site/catalytic site of rRsbW shows higher denaturant sensitivity than the tentative dimerization region of this enzyme.

## Introduction

RsbW, a cytoplasmic protein, is expressed by *Staphylococcus aureus* and many other Gram-positive bacteria [1–3]. Nearly 55–85% identity was noticed among the RsbW protein sequences from different bacterial species. The operon that encodes RsbW in *S*. *aureus* also produces many other proteins including RsbU, RsbV, and SigB [1–3]. Proteins orthologous to *S*. *aureus* RsbU, RsbV, and SigB are expressed by *Staphylococcus epidermidis*, *Bacillus subtilis*, and *Listeriamonocytogenes* as well. SigB, an alternative sigma factor, usually controls the gene expression under the conditions of environmental stress [1–3]. In addition, SigB is also involved in the pathogenesis caused by *S*. *aureus*, *S*. *epidermidis*, and *L*. *monocytogenes*[2–5]. Of the Rsb proteins, RsbW proteins are usually composed of ~157–161 amino acid residues, exist as the dimers in the aqueous solution, blocks the SigB-mediated transcription, and causes phosphorylation of a Ser residue in RsbV [1–3, 6–9]. The physical interaction between RsbW and RsbV or SigB was shown by both *in vitro* and *in vivo* probes [1–3, 6, 8, 10, 11]. The binding stoichiometry of RsbW to SigB is 2:1 and that to RsbV is 2:2 [8]. The affinity of RsbW to RsbV is higher than that to SigB, indicating the preferential formation of a complex between RsbW and the dephosphorylated RsbV [8, 10]. Collectively, RsbV performs as an anti-anti-sigma factor, whereas, RsbW works both as an anti-sigma factor and a serine kinase. Conversely, RsbU acts as a phosphatase and is involved in the dephosphorylation of phosphorylated RsbV [1–3, 12]. The Rsb proteins presumably control the activity of SigB by a similar manner in the low GC Gram-positive bacteria [1–3, 7, 12]. In the absence of stress response, the phosphorylation of RsbV by RsbW dislodges the latter from the former. The freely available RsbW then makes a complex with SigB that in turn blocks the activity of the latter protein. In the presence of stress response, removal of the phosphate group from the phosphorylated RsbV by RsbU makes the SigB available for transcription as the dephosphorylated RsbV forms a stronger complex with RsbW.

A newly-synthesized polypeptide chain needs to be folded correctly prior to performing any biological work in the living cell. The equilibrium unfolding studies of many proteins have indicated that these macromolecules are folded either by a two- or by a higher-state mechanism [13, 14]. While a two-state folding occurs without the synthesis of an intermediate, a three- or a higher-state folding proceeds through the generation of one or more intermediates. The unfolding studies are also widely used to understand about the thermodynamic stabilization of a protein in the presence of the cognate ligand or mutation [15–19]. In addition, unfolding studies are extensively exploited in understanding abnormal protein folding in various diseases, developing nanomaterials useful for tissue repair/engineering, preparing medically important enzymes and hormones, producing recombinant proteins with increased storage tolerance, and in screening new drug molecules [18, 20–24].

RsbW shares nearly 27% identity with *B*. *subtilis* SpoIIAB at the amino acid sequence level [1]. SpoIIAB, an inhibitor of SigF, is a single-domain protein, and dimerizes using its N-terminal end resides [25]. Currently, the domain structure of RsbW or the way it forms dimers in the aqueous solution is not clearly known. Additionally, the relationship between the function and dimeric state of the RsbW proteins and their folding-unfolding mechanism in the presence and absence of SigB have not been investigated yet using any denaturant. The resulting data mayexpedite the screening of theinhibitors of both SigB and kinases involved in bacterial pathogenesis [26, 27]. In the current study, we have thoroughly investigated the structure, function, and unfolding of *S*. *aureus* RsbW using a recombinant variant (rRsbW) of this protein. Our data show that rRsbW is composed of a single-domain and unfolds via the formation of at least two intermediates in the presence of urea or guanidine hydrochloride. The properties of no intermediate completely matched with those of others and the native rRsbW. In addition, different regions of rRsbW have shown different sensitivity to both denaturants.

## Materials and methods

### Materials

Acrylamide, ANS (8-anilino-1-napthalene sulfonate),antibodies (alkaline phosphatase-linked goat anti-mouse and anti-his antibody), ATP (adenosine triphosphate), bisacrylamide, chymotrypsin, DNA gel extraction kits, DNA marker, enzymes (*Eco*RI, *Hind*III, *Nco*I, *Xho*I, T4 DNA ligase, etc.), glutaraldehyde, guanidine hydrochloride (GdnCl),isopropyl β-D-1-thiogalactopyranoside(IPTG), kanamycin, Ni-NTA resin, oligonucleotides, plasmid purification kit, phenylmethane sulfonylfluoride (PMSF), protein marker, proteinase K, trypsin, and ureawere purchased from Sigma, Qiagen, GE Healthcare Bioscience Ltd., Fermentas, SRL, and Hysel India Pvt Ltd.*S*. *aureus* Newman was obtained as a gift from Prof. C. Y. Lee, the University of Arkansas for Medical Sciences. Plasmid pET28a and *Escherichia coli* BL21(DE3)were donated by late Prof. P. Roy, Bose Institute.

### Growth of different bacteria

We have regularly cultivated *E*. *coli*BL21(DE3)]and *S*.*aureus* Newman in Luria-Bertani (LB) medium [28] and in trypticase soy broth [29], respectively. Bacterial strains harboring particular plasmids were grown in the media supplemented with appropriate antibiotics.

### Basic molecular biological experiments

The basic molecular biological methods such as digestion of DNA by restriction endonuclease, ligation of DNA by T4 DNA ligase, agarose gel electrophoresis, DNA transformation, sequencing of DNA inserts, native PAGE, SDS-PAGE, gel staining, Western blotting, DNA purification, DNA estimation, and polymerase chain reaction (PCR) were performed as described [28, 30, 31]. We estimated the content of total protein by the Bradford assay using bovine serum albumin as reported earlier [31]. The molar concentration of rRsbW and a recombinant *S*. *aureus* RsbV (rRsbV) were determined using the molecular mass of the respective monomer. The chromosomal DNA from *S*. *aureus* Newman was purified by a standard procedure [32].

### Protein purification

To purify rRsbV, a part of *S*. *aureus* Newman genomic DNA was amplified using primers RsbV1 (5’CGCCCCATGGCTAATCTTAATATAGAAACAAC3’) and RsbV2 (5’CCACTCGAGTTCGACCTCCGTTCCTTC3’) as described [31]. The resulting 333 bp DNA insert was cloned into pET28a as stated earlier [31]. The generated plasmid that bears no mutation in the cloned DNA fragment was named p1412 (data not shown). Plasmid p1412 was transformed into *E*. *coli* BL21 (DE3) to generate SAU1412.*E*. *coli* strain SAU1412 was grown to the OD_590_ of 0.5 at 37°C. The cells were induced with 200 μM of IPTG for 12–16 h at 16°C. The induced cells were sequentially harvested, washed with 0.9% NaCl and sonicated to make cell extract. rRsbV was purified from the cell extract by similar Ni-NTA chromatography as described before [31].

To purify rRsbW, a 480 bp DNA insert was amplified using *S*. *aureus* Newman genomic DNA and primers rsbWF (5’CGGAATTCATGCAATCTAAAGAAG3’) and rsbWR (5’CCCAAGCTTAGCTGATTTCGACTC3’) as stated [31]. A plasmid, namely p1414, was constructed by cloning the above DNA fragment to pET28a by a standard method [31]. As evident from DNA sequencing, there was no mutation in the DNA fragment ofp1414 (data not shown). An *E*. *coli* strain, designated SAU1414, was generated by transforming p1414DNA into *E*. *coli* BL21 (DE3). rRsbW was purified from the SAU1414 cell extract by a similar Ni-NTA chromatography as described above. The eluted rRsbV and rRsbW were dialyzed against a buffer A [50 mM Tris HCl (pH 8.0), 50 mM KCl,2 mM MgCl_2_and 5% glycerol] or a buffer B [20 mM Phosphate buffer (pH 8.0), 100 mM NaCl, and 5% glycerol] prior to their use.

### Enzymatic activity

To check whether rRsbV and rRsbW are functional, we have carried out a serine kinase assay by a standard procedure [9] with minor modifications. Briefly, rRsbW (2.5 μg) in buffer A was incubated with the equal amount of rRsbV for 5 min at room temperature. ATP (1 or 5 mM) was added to the reaction mixture followed by its incubation for additional 5–15 min at the same temperature. After adding a loading dye [36% glycerol and 0.3% bromophenol blue], the reaction mixture was analyzed by a native-12% PAGE. The image of the Coomassie brilliant blue (R250)-stainedgel was captured by a Gel Doc^™^ XR+ (Bio-Rad) system. A densitometric analysisof the gel picture was performed using Image Lab^™^ software (Bio-Rad). The intensity values of the protein bands were determined from the resulting scanned data.

### Limited proteolysis

To know about the domain structure and the flexible region of rRsbW, we have performed limited proteolysis of this protein with chymotrypsin, trypsin, and proteinase K using the standard procedures as described before [31]. In short, rRsbW (10 μM) in buffer B was digested with a proteolytic enzyme for 64 min at 25°C. At regular time intervals, an aliquot (50 μl) was transferred from the reaction mixture to a centrifuge tube containing an SDS sample buffer. After incubating the aliquots at 100°C for 2 min, they were analyzed by an SDS-13.5% PAGE. The picture of the stained polyacrylamide gel was obtained as stated above. Toidentify the proteolytic cleavage sites, the N-terminal ends of major rRsbW fragments were sequenced as reported [31].

### Biochemical and biophysical analyses

To acquire hints about different structures of rRsbW, the far-UV Circular Dichroism (CD)spectrum (200–260 nm),near-UV CD spectrum (250–310 nm), and the intrinsic Tyr fluorescence spectrum (λ_ex_ = 280 nm and λ_em_ = 290–340 nm) of this protein in buffer B were recorded at 25°C as reported [31, 33, 34]. Protein concentration used in far-UV CD or Tyr fluorescence spectroscopy was 10μM, whereas, that for near-UV CD was40 μM. The cuvette path lengths for far-UV CD and near-UV CD were kept 1 mm and 5 mm, respectively. Each CD or Tyr fluorescence intensity value was corrected by deducting the buffer value from the value of buffer containing rRsbW. To obtain clues about the amount of secondary structural element in rRsbW, its far-UV CD spectrum was analyzed by CDNN software [35].

To know about the hydrophobic surface of rRsbW, the ANS fluorescence spectrum (λ_ex_ = 360 nm and λ_em_ = 400–600 nm) of this protein (10 μM) in buffer B was recorded at 25°C as demonstrated earlier [31, 34]. The concentration of ANS in buffer was kept 100 μM. Each ANS fluorescence intensity value was corrected by subtracting the buffer value from the value of buffercontaining rRsbW.

To determine the oligomeric status of rRsbW (10 μM),we have performed the glutaraldehyde-mediated cross-linking experiment as mentioned earlier [31]. Briefly, protein in buffer B was exposed to 0.1% glutaraldehyde solution for 2 min at 25°C. After termination of reaction with an SDSsample buffer [28], reaction mixtures were boiled for 2 min followed by their analysis by an SDS-13.5% PAGE. The documentation and thedensitometric analysis of gel picturewere carried out as described above.

To learn about the shape and size of rRsbW, analytical gel filtration chromatography was carried out as stated [31]. In brief, an aliquot (20 μM)of rRsbW in buffer B was eluted at the flow rate of 1 ml/min using a Superdex 200 column. Some marker proteins (e.g. RNaseA, Ovalbumin, Conalbumin, and Carbonic anhydrase), and blue dextran in buffer B were also separately eluted using same column and conditions. The retention factor of each protein was determined using its elution volume, the total volume of thecolumn, and theelution volume of blue dextran. The plot of retention factors of marker proteins versus their molecular mass was used to calculate the molecular mass of rRsbW.

### Probing unfolding and refolding of rRsbW

To study the unfolding of rRsbW, this protein (10–20 μM) was incubated with 0–7 M urea or 0–5 M GdnCl for 16–18 h at 4°C as reported [31]. Aliquots of the freshly prepared stock solution of urea (10 M) or GdnCl (8 M) were always used for treatment. To determine the effects of denaturants on the different structures of rRsbW, the far-UV CD, near-UV CD, and intrinsic Tyr fluorescence spectra of urea- or GdnCl-treated rRsbW aliquots were recorded individually as described above. To find out the hydrophobic surface areas of rRsbW in the presence of urea or GdnCl, the ANS fluorescence spectra of urea- or GdnCl-exposed rRsbW samples were also recorded as mentioned above. The spectroscopic signals were corrected by deducting the buffer values from the values of buffer containing protein.

To study the refolding of rRsbW, it was denatured with 7 M urea or 5 M GdnCl by a method similar to that stated above. To remove the denaturants, each unfolded protein was dialyzed against the protein-containing buffer for 16–18 h at 4°C. The far-UV CD and Tyr fluorescence spectra of native, refolded, and denatured proteins were recorded as stated above. Protein concentrations of native, denatured and refolded proteins were kept identical during the spectroscopic study. To check whether the refolded rRsbW is functional, its serine kinase assay was performed as demonstrated above.

The values of urea/GdnCl concentration at the midpoint of unfolding transition (*C*_m_) were estimated by nonlinear fitting of the unfolding curves to the two- or three-state equation using GraphPad Prism (GraphPad Software Inc.) as stated earlier [13, 31].

To find out the effects of denaturants on the dimeric rRsbW, we have incubated the urea- or GdnCl-treated rRsbW aliquots (each 10 μM)with glutaraldehyde and the resulting reaction mixtures were analyzedasreported above. To better understand the effects of denaturants on the shape of rRsbW, the urea- or GdnCl-exposed rRsbW aliquots (each 20 μM) were analyzed by an analytical gel filtration chromatography as stated above.

### Homology modeling

To develop a three-dimensional structural model of *S*. *aureus* RsbW, a suitable template was searched by Swiss-Model (http://ExPasy.org) using the amino acid sequence of this regulator. Of the probable templates, the crystallographic structure of *Geobacillusstearothermophilus* SpoIIAB (PDB ID: 1TH8) [25] was adjudged to be the best (*E* value: 1e^-11^). SpoIIAB, an anti-sigma factor, shows ~33% identity with that of *S*. *aureus* RsbW at the sequence level. A model structure of dimeric RsbW was produced by Swiss-Model using 1TH8 as the template. The model structure was visualized using PyMol (http://PyMol.org).

## Results

### Purification and properties of rRsbW

**Fig 1:**
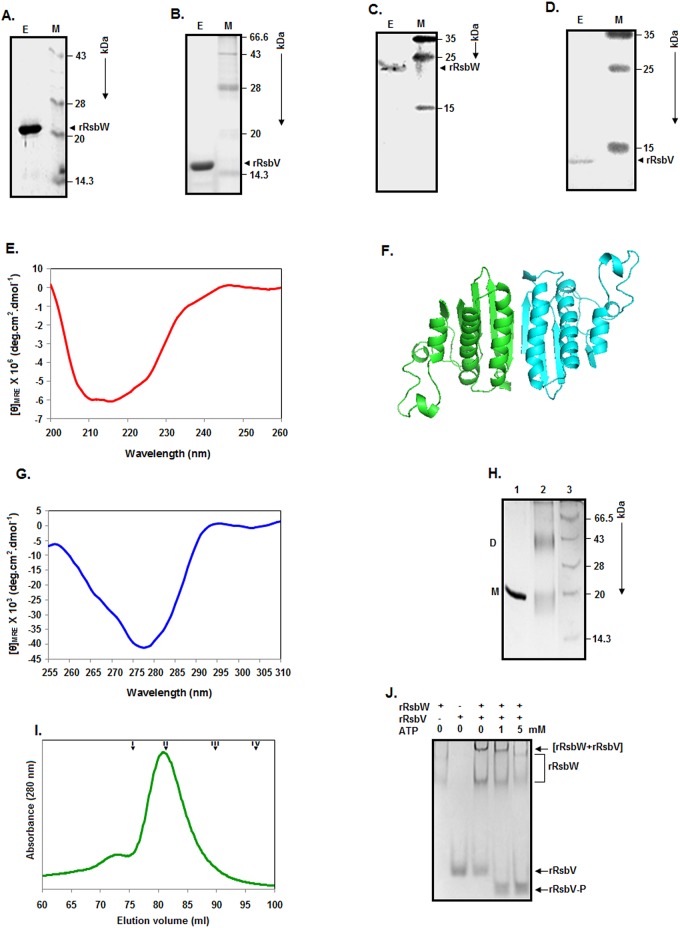
Purification and properties of proteins. Different elution fractions collected during protein purification were analyzed by SDS-13.5% PAGE. (A, B) The rRsbW- and rRsbV-specific protein bands are shown.(C, D) Western blot analysis of the elution fractions carrying rRsbW and rRsbV. The molecular masses (in kDa) of the marker proteins (M) are indicated on the right side of the gel/blot pictures. (E) Far-UV CD spectrum of rRsbW. (F) Three-dimensional structural model of RsbW. (G) Near-UV CD spectra of rRsbW. (H) Chemical crosslinking of rRsbW. The glutaraldehyde (GCHO)-treated (lane 2) /untreated (lane 1) protein aliquots were analyzed by an SDS-13.5% PAGE. The molecular masses (in kDa) of the marker proteins (lane 3) bands are shown on the right of the gels. ‘D’ and ‘M’ indicate rRsbW dimer and monomer, respectively. (I) Gel filtration chromatography of rRsbW by a standard method as described [31]. I, II, III, and IV denote the peak positions of Conalbumin (75 kDa), Ovalbumin (43 kDa), Carbonic anhydrase (29 kDa), and RNase A (13.7 kDa), respectively. (J) Serine kinase assay. The reaction mixtures carrying rRsbW and or rRsbV, and the indicated amount of ATP were incubated followed by their analyses using a native PAGE as stated in the Materials and Methods. The rRsbW, rRsbW-rRsbV complex, rRsbV, and the phosphorylated rRsbV are shown at the right side of gel picture.

To elaborately study the structure, function, and the folding-unfolding mechanism of RsbW proteins, a recombinant *S*. *aureus* RsbW (rRsbW) was purified to near homogeneity using Ni-NTA chromatography (Fig 1A). rRsbW was constructed by linking a stretch of six His residues and thirty other residues at the N-terminal end of RsbW (UniProt Code:A6QIR1), added to circumvent problems faced in cloning. A recombinant *S*. *aureus* RsbV (rRsbV), a putative substrate of the rRsbW kinase, was also purified by similar affinity chromatography (Fig 1B). rRsbV was created by joining the C-terminal end of RsbV (UniProt Code: A0A0H3K9P0) with additional eight amino acid residues including a stretch of six His residues. The theoretical molecular masses of rRsbV and rRsbW, determined from their respective sequences using a software program (web.espaxy.org/portparam), are 13.34 kDa and 21.74 kDa, respectively. The gel pictures (Fig 1A and 1B) reveal that the experimental molecular masses of rRsbW and rRsbV are close to these calculated masses. Both rRsbW (Fig 1C) and rRsbV (Fig 1D) also interacted with an anti-his antibody, confirming the presence of polyhistidine tags in these proteins.

To know about the secondary structure of rRsbW, the far-UV CD spectrum of this protein was recorded as stated in the Materials and Methods. The spectrum shows an extended peak at ~210 to 220 nm, indicating the presence of α-helix in rRsbW (Fig 1E). Analysis of the spectrum by CDNN [35] suggests that there is nearly 22% α-helix, 21.5% β-sheet, and 39% coil in rRsbW. The putative structure of *S*. *aureus* RsbW (Fig 1F), developed using a geobacillus anti-sigma factor [25], also recommends that this protein may be composed of 34% α-helix and 24% β-sheet.

The near-UV CD spectrum of a protein is usually originated from its resident aromatic amino acid residues, disulfide bonds and bound cofactors [36]. Such spectrum is very useful to understand the three-dimensional structure of a protein. rRsbW carries no Trp residue but harbors seven Phe and eight Tyr residues at various locations. While Phe is located at positions 36, 43, 66, 108, 126, 146, and 155, Tyr is situated at positions 54, 73, 95, 107, 128, 138, 167, and 179, respectively. To experimentally gain some clues about the tertiary structure of rRsbW, the near-UV CD spectrum of this protein was recorded as mentioned in Materials and Methods. The spectrum of rRsbW shows a peak of negative ellipticity at ~275–280 nm (Fig 1G). Phe and Tyr usually yield peaks at ~255–270 nm, and ~275–282 nm, respectively [36]. The peak of the rRsbW spectrum, therefore, may be a Tyr-specific peak. The reason as to why the Phe-specific peak is absent is not known with certainty.

A recombinant *S*. *aureus* RsbW with a size biggerthan rRsbW previously formed dimers in the aqueous solution [7]. To see whether rRsbW also forms the dimers in solution, a glutaraldehyde-mediated crosslinking experiment of this macromolecule was performed as stated above. There is a formation of ~43 kDa protein species in the presence of glutaraldehyde, suggesting the dimerization of rRsbW in the aqueous solution (Fig 1H). To confirm the crosslinking data, rRsbW was analyzed by gel filtration chromatography as described. The rRsbW chromatogram primarily shows a single peak with the retention volume of ~80.89 ml (Fig 1I). In comparison with the elution volumes of some monomeric proteins (Fig 1I), the elution volume of rRsbW indicates that its apparent molecular mass is54.57 kDa. As the theoretical mass of rRsbW is 21.74 kDa, this protein mostly exists as a dimer in the aqueous solution. The somewhat early elution of dimeric rRsbW might have occurred due to its elongated shape.

To confirm that the additional residues have negligibly affected the biological activity of RsbW, a serine kinase assay in the presence and absence of ATP was performed using rRsbV as demonstrated above. Analysis of the reaction mixtures by a native PAGE reveals the formation of phosphorylated rRsbV in the presence of ATP only (Fig 1J), indicating that rRsbW can act as a serine kinase despite carrying additional amino acid residues.

### Domain structure of rRsbW

**Fig 2:**
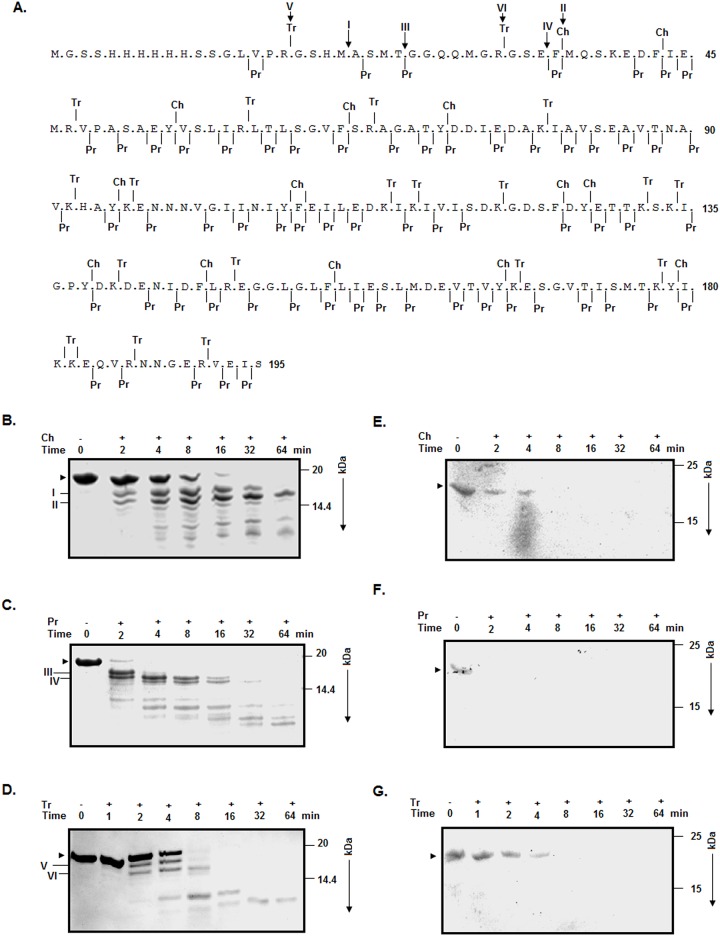
Limited proteolysis of rRsbW. (A) The sequence of rRsbWwith the cleavage sites of chymotrypsin (Ch), proteinase K (Pr), and trypsin (Tr). All of the predicted cut sites, determined using a computational tool [31], are presented by verticle lines on both sides oftheamino acid sequence. The experimentally-detected cut sites (I-VI) are shown by downward arrows over the rRsbW sequence. The polyhistidine tag carrying regionofrRsbW is composed of amino acid residues 1 to 36. (B, C, D) Analyses of the rRsbW fragments by SDS-13.5% PAGE after Ch, Pr, and Tr digestions, respectively. Arrowhead and I−VI denote the intact rRsbW and its major proteolytic fragments of rRsbW, respectively. (E, F, G) Western blotting analyses of the Ch -, Pr -, and Tr -digested rRsbW fragments using an anti-his antibody. The molecular masses (in kDa) of marker proteins are shown at the right side of the gel/blot pictures.

The retention of both the structure and function (Fig 1) in rRsbW indicates that it could be used as a model for obtaining additional clues about the RsbW proteins. Our modeling and other computational investigations suggest that RsbW monomer may be composed of only one domain (Fig 1 and data not shown). To verify the hypothesis, limited proteolysis [37] of rRsbW was separately performed with chymotrypsin, trypsin, and proteinase K as described [31]. These enzymes were selected as they have more than ten predicted cut sites along the entire length of rRsbW (Fig 2A). However, the majority of the cut sites will not be recognized by the above proteolytic enzymes if rRsbW truly carries one domain. Fig 2B reveals the generation of fragments I and II from rRsbW at the very early stage of its digestion with chymotrypsin. Of the fragments, fragment I remained somewhat stable even at the late stage of digestion with chymotrypsin. Many smaller fragments were also produced at the late stage of digestion. Digestion of rRsbW with proteinase K produced two major fragments (III and IV) and two minor fragments at the early stage(Fig 2C). Unlike fragment IV, fragment III was degraded very fast upon further continuation of digestion. Like chymotrypsin, cleavage of rRsbW with trypsin also made two fragments (V and VI) at the early stage (Fig 2D). None of the proteolytic fragments has shown a reaction with the anti-his antibody though it is seen with rRsbW (Fig 2E–2G), suggesting the removal of polyhistidine tag from rRsbW by all of three enzymes used here.

To correctly map the cut sites in rRsbW, we have determined the N-terminal end residues of major proteolytic fragments occurring within the first two minutes of digestion. The five N-terminal end residues of fragments I, II, III, IV, V, and VI are ASMTGG, MQSKED, GGQQM, FMQSK, GSHMA, and GSEFM, respectively. Thatis, all these sites are in the N-terminally-located, polyhistidine tag carrying region of rRsbW (Fig 2A). As the molecular masses of the above fragmentsrange from ~14.65 to ~17.17 kDa, they are smaller than native RsbW (17.92 kDa), indicating that the extreme C-terminal end of rRsbW is also sensitive to the proteolytic enzymes. A comparative analysis of the masses of the proteolytic fragments suggeststhattherelatively enzyme-resistant region of rRsbW is composed of amino acid residues ~37–175. Taken together, rRsbW proteins may have only one domain.

### Urea-induced unfolding of rRsbW

**Fig 3:**
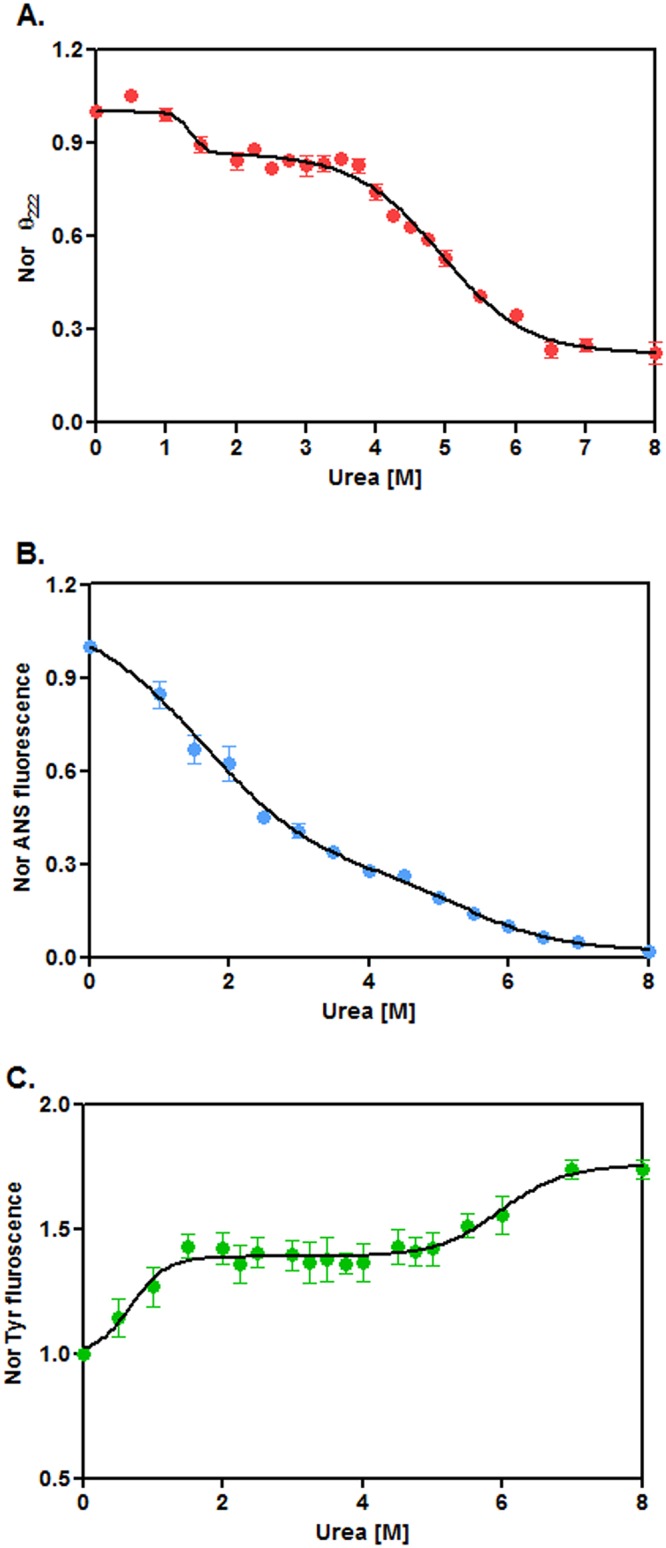
Urea-induced unfolding of rRsbW. (A) The ellipticity values of rRsbW at 222 nm (θ_222_), were derived from their far-UV CD spectra (S1 Fig), normalized (Nor), and plotted versus the corresponding urea concentrations. The ANS fluorescence intensity values at 480 nm (B) and the intrinsic Tyr fluorescence intensity values at 308 nm (C) were obtained from the corresponding spectra of rRsbW (S1 Fig) were plotted similarly.

To obtain clues about the folding-unfolding mechanism of rRsbW, we have separately recorded the far-UV CD, ANS fluorescence, and intrinsic Tyr fluorescence spectra (S1 Fig) of this enzyme in the presence of 0–8 M urea using standard procedures [31]. A biphasic curve was obtained when the ellipticity values of rRsbW at 222 nm were plotted against the corresponding urea concentrations (Fig 3A). The curve shows the transitions at ~1–1.75 and ~3.75–6.5 M urea, respectively. A non-sigmoidal curve was also produced when the ANS fluorescence intensity values of rRsbW at 480 nm were plotted against the equivalent urea concentrations (Fig 3B). The plotting of intrinsic Tyr fluorescence values of rRsbW(at 308 nm)against the matching urea concentrations yielded the curve with two transitionsat ~0.25–1.5 and ~4.75–7 M urea, respectively(Fig 3C). Taken together, the urea-induced unfolding of rRsbW most possibly proceeded via the formation of some intermediate.

To check the reversibility of the above urea-induced unfolding, unfolded form of rRsbW was gradually refolded followed by the recording of its CD and Tyr fluorescence spectra as described above. Similar spectra of unfolded and native forms of rRsbW were also recorded for comparison. The results show the complete overlapping of the CD spectrum and the Tyr fluorescence spectrum of refolded protein with those of native form (S2 Fig). To check whether the refolded rRsbW retains any serine kinase activity, a suitable assay was performed as stated above. Separation of the resulting proteins by a native PAGE shows the formation of phosphorylated rRsbV in the presence of ATP and refolded rRsbW (S2 Fig). Such structural and functional restoration indicates that the urea-induced unfolding of rRsbW is fully reversible under the conditions of the study.

To better understand the mechanism of urea-induced unfolding, the unfolding data of rRsbW(Fig 3) were analyzed by different unfolding models [13, 31]. Each unfolding curve of rRsbW showed best fitting to a three-state model. The CD data yielded the *C*_m_ values of 1.36±0.17M and 4.93±0.06 M urea, whereas, ANS fluorescence resulted in the *C*_m_ values of 1.62±0.21M and 5.44±0.63 M urea, respectively. On the other hand, the *C*_m_ values, determined from the Tyr fluorescence data, were 0.69±0.13 M and 5.96±0.2 M urea, respectively. Collectively, a rRsbW intermediate seems to be populated mostly at ~2.5–3.5 M urea.

### Properties of urea-made rRsbW intermediates

**Fig 4:**
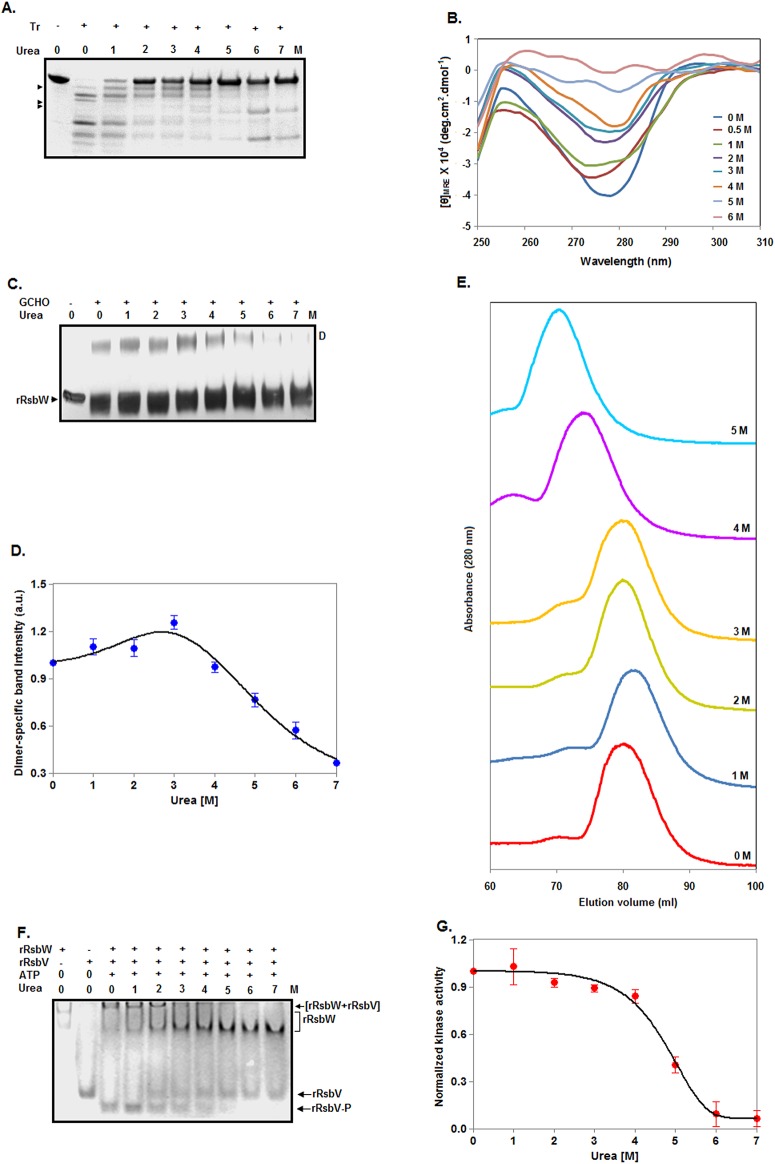
Characterization of intermediates made by urea. (A) Analyses of proteolytic fragments of rRsbW by SDS-13.5% PAGE. Protein aliquots, pre-equilibrated with 0–7 M urea, were digested with (+)/without (-) trypsin for 8 min prior to gel analysis. Arrowheads indicate either new protein fragments or the fragments with a comparatively increased intensity at 1–4 M urea. (B) Near-UV CD spectra of equimolar concentrations of rRsbW at the indicated concentrations of urea. (C) Crosslinking of 0–7 M urea-treated rRsbW with (+)/without (-) glutaraldehyde (GCHO). The crosslinked molecules were separated by SDS-13.5% PAGE. ‘D’ indicates dimeric rRsbW. (D) The dimer-specific band intensity values were estimated from panel C and plotted versus the corresponding urea concentrations. (E) Gel filtration chromatography of rRsbW at 0–5 M urea. (F) Kinase assay. The assay was performed using the 0–7 M urea-exposed rRsbW, rRsbV, and 1 mM ATP for 10 min at room temperature. The reaction mixtures were analyzed by a 12% native PAGE. The protein bands corresponding to rRsbW, rRsbW-rRsbV complex, phosphorylated rRsbV, and non-phosphorylated rRsbV are indicated. The phosphorylated rRsbV bands were scanned to determine their intensity values at 0–7 M urea. Considering that the extent of rRsbV phosphorylation at 0 M urea corresponds to 100% kinase activity, kinase activities of rRsbW at 1–7 M urea were determined. After normalization, the kinase activity values were plotted against the corresponding urea concentrations (G).

To validate the synthesis of the rRsbW intermediate, trypsinolysis of rRsbW was performed at 0–7 M urea as demonstrated [31]. Fig 4A reveals that the proteolytic patterns of rRsbW in the presence and absence of urea are not identical. There is the production of additional protein fragments from rRsbW at ~1–4 M urea, confirming the generation of rRsbW intermediate at these urea concentrations. The near-UV CD spectra show the gradual decrease of the ellipticity values of rRsbW (Fig 4B) at 280 nm upon increasing the urea concentration from 0 to 6 M. We observed nearly 50% reduction of the ellipticity values at 2–4 M urea, indicating that intermediate retains sufficient extent of tertiary structure at these urea concentrations and is not a molten globule [38].

To know about the oligomeric status of intermediate, a glutaraldehyde-mediated crosslinking experiment of rRsbW was performed in the presence of 0–7 M urea. The gel picture shows the drastic reduction of the dimer-specific rRsbW band intensity at the urea concentrations of higher than 4 M (Fig 4C). The plot of dimer-specific band intensity versus the corresponding urea concentrations reveals the retention of more than 95% dimeric rRsbW at 4 M urea (Fig 4D). Currently, the reason for the formation of relatively higher extent of dimeric rRsbW at ~2–3 M urea is not clearly known. However, the data indicate that the rRsbW intermediate exists as the dimeric molecules in the aqueous solution. To substantiate the suggestion, a gel filtration chromatography of rRsbW was carried out in the presence of 0–5 M urea. Each sample primarily resulted in a single peak with a discrete retention volume (Fig 4E). The elution volumes of 0, 1, 2, 3, 4, and 5 M urea-treated rRsbW were 80.40, 81.88, 80.25, 80.37, 74.43, and 70.75 ml, respectively. The elution profiles though not yielding apparent molecular masseshave provided valuable clues about the shape of rRsbW in the presence of urea. The 0, 2, and 3 M urea-treated samples have nearly similar elution volume, suggesting little or very little changein the shape of rRsbW at 2–3 M urea. Conversely, ~6–10 ml smaller elution volume indicates some swelling of native rRsbW at 4–5 M urea. In sum, the proposed rRsbW intermediate formed at ~2.5 to 3.5 M urea may have a native rRsbW-like shape. Additionally, ~1.5 ml higher elution volume suggests that the shape of rRsbW at 1 M urea is smaller than that of native rRsbW. At ~0.5–1 M urea, the extent of secondary structure(Fig 3A) of rRsbW remained nearly unaltered, whereas, the amounts of the hydrophobic surface area (Fig 3B) or tertiary structure (Fig 4B) of this protein were reduced by ~15% and ~20%, respectively. Thus, the structure and hydrophobic surface area of rRsbW at ~0.5–1 M urea neither matchthose of ~2.5–3.5 M rRsbW intermediate (mentioned above) nor native rRsbW. Hence, there might be the formation of an additional rRsbW intermediate at ~0.5–1 M urea.

To check the functionality of the urea-generated rRsbW intermediates, we have performed a serine kinase assay in the presence and absence of urea. Fig 4F shows the formation of phosphorylated rRsbV by rRsbW in the presence of 0–5 M urea. At urea concentrations of higher than 5 M, there is almost no phosphorylation of rRsbV. Using the extent of phosphorylatedrRsbVat 0 M urea as the measure of thefull kinase activityof rRsbW,we have determined its activities in the presence of urea. The data show almost no change of serine kinase activity at 0–1 M urea (Fig 4G). Conversely, there is about ~10–15% loss of kinase activity at 2–4 M urea. Control experimentsreveallittle or very little changein thestructure of the substrate(rRsbV)of serine kinase during the period of theassay in the presence of 0–7 M urea (S1 Fig). In summary, both rRsbW intermediates (stated above) possess >85% serine kinase activity.

### GdnCl-induced unfolding of rRsbW

**Fig 5:**
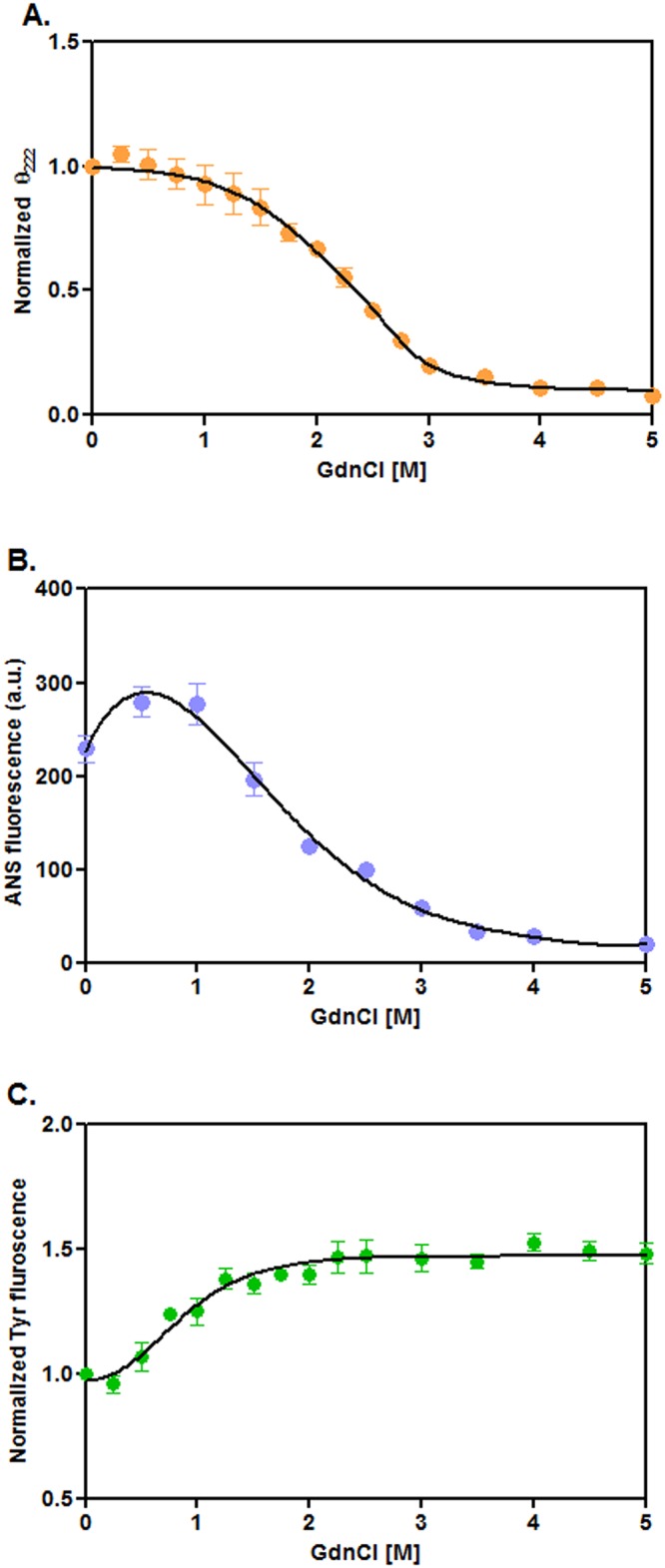
GdnCl-induced unfolding of proteins. (A) The θ_222_values of rRsbW,were extracted (from S3 Fig), normalized, and plotted versus the equivalent GdnCl concentrations. The ANS fluorescence (B) /intrinsic Tyr fluorescence intensity (C) values at 480/308 nm were derived(from S3 Fig) and similarly plotted.

Many proteins areunfolded by different mechanisms in the presence of different denaturants [31, 39, 40]. rRsbW may also be unfolded by a distinct mechanism in the presence of a denaturant other than urea. To verify it, we have separately recorded the far-UV CD, ANS fluorescence, and intrinsic Tyr fluorescence spectra (S3 Fig) of rRsbW in the presence of 0–5 M GdnCl as described [31]. The plotting of the ellipticity values of rRsbW (at 222 nm)against the related GdnCl concentration has yielded a curve that is not truly sigmoidal in nature (Fig 5A). There is greater than 70% loss of ellipticity values at ~1–3 M GdnCl. The curve obtained by plotting the ANS fluorescence intensity values of rRsbW (at 480 nm)against the correspondingGdnCl concentrations was also not sigmoidal in nature (Fig 3B). However, the intrinsic Tyr fluorescence values of rRsbW(at 308 nm) against the equivalent GdnCl concentrations appear to be monophasic in nature with a transition at ~0.25–1.5 M GdnCl(Fig 5C). Jointly, the GdnCl-induced unfolding might also have occurred via the generation of at least one rRsbW intermediate.

To ensure that the GdnCl-induced unfolding of rRsbW follows a reversible pathway, we have recorded the CD and Tyr fluorescence spectra of native, refolded, and unfolded forms of this regulator as stated above. The data show the complete superimposition of the CD spectrum or the Tyr fluorescence spectrum of native protein with those of refolded rRsbW (S4 Fig). To test the presence of kinase activity in the refolded rRsbW, we have performed an appropriate assay using rRsbV as demonstrated above. Analysis of the resulting reaction mixtures by a native PAGE reveals the production of phosphorylated rRsbV in the presence of refolded rRsbW and ATP (S4 Fig). In sum, the GdnCl-induced unfolding of rRsbW is also completely reversible in nature.

To obtain clues about the mechanism of GdnCl-induced unfolding, we have analyzed the relevant unfolding data of rRsbW(Fig 5) using different unfolding models [13, 31]. The CD-specific unfolding curve of rRsbW shows the best fitting to the three-state model that has resulted in the *C*_m_ values of 2.11±0.29M and 2.68±0.24 M GdnCl, respectively. Conversely, the Tyr fluorescence-specific curve fits best to the two-state model that has yielded the *C*_m_ value of 0.84±0.08M GdnCl. On the other hand, the ANS fluorescence data could not be fitted either to the two-state or the three-state modelas the ANS fluorescence intensity value at 0.5–1 M GdnCl was nearly 20% higher than that at 0 M GdnCl. Together, the GdnCl-induced unfolding of rRsbW most possibly occurred via four states with three steps and twointermediates. While the first intermediate was perhaps populated at ~0.5–1 M GdnCl, the second intermediate might have been formed at ~2.3 M GdnCl.

### Properties of GdnCl-made intermediates

**Fig 6:**
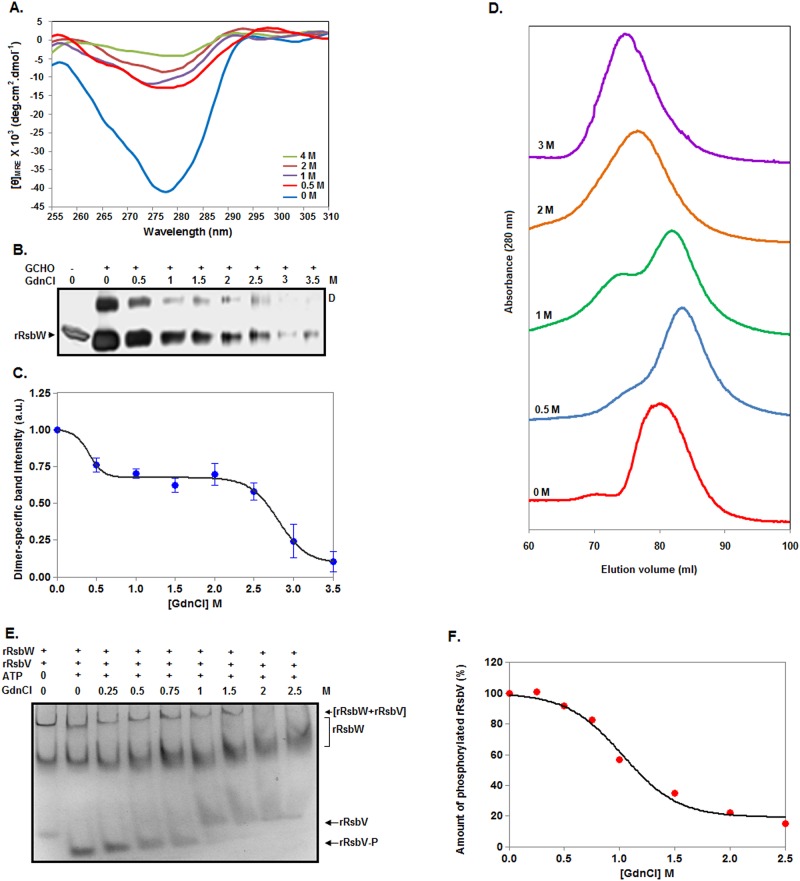
Characterization of intermediates produced by GdnCl. (A) Near-UV CD spectra of rRsbW at 0–4 M GdnCl. (B) SDS-13.5% PAGE analysis of 0–3.5 M GdnCl-exposed rRsbW molecules crosslinked with (+)/without (-) glutaraldehyde (GCHO). (C) The plot of dimer-specific band intensity versus the matching GdnCl concentrations. The dimer-specific band intensity values were estimated from panel B as demonstrated above. (D) Gel filtration chromatography of rRsbW at 0–3 M GdnCl. (E) Kinase assay. The assay was carried out using a similar method as stated in Fig 4F. Only urea-treated rRsbW was replaced with the indicated GdnCl-treated rRsbW. (F) The plot of kinase activity versus equivalent GdnCl concentrations. The plot was made using the intensity of phosphorylated rRsbV bands as stated in Fig 4F.

To determine the properties of the rRsbW intermediates, we have recorded the near-UV CD spectra of rRsbW at 0–4 M GdnCl as described [31]. Fig 6A reveals that there is nearly 70–90% reduction of the ellipticity values of rRsbW at 280 nm upon raising the GdnCl concentration from 0 to 0.5 M and higher. The data suggest that both intermediates have lost the majority of their tertiary structures at 0.5 M and higher GdnCl concentrations. Of the intermediates, the intermediate generated at ~0.5–1 M GdnCl may have a molten globule-like structure [38] as it is composed of very little tertiary structure, sufficient extent of secondary structure (Fig 5A) and an increased amount of hydrophobic surface area (Fig 5B).

To know about the oligomeric state of intermediates, we have performed a glutaraldehyde-mediated crosslinking experiment of rRsbW in the presence of 0–3.5 M GdnCl. The gel picture shows a rapid loss of the dimeric-specific rRsbW band intensity at GdnCl concentrations of >2.5 M (Fig 6B). The plot of dimer-specific band intensity versus the related GdnCl concentrations shows about 25–35% reduction of the dimeric-specific rRsbW band intensity at 0.5–2.5 M GdnCl (Fig 6C). The results suggest that both rRsbW intermediates exist as the dimers in the aqueous solution. To prove the proposal, we have carried out a gel filtration chromatography of rRsbW at 0–3 M GdnCl. The 1 M GdnCl-treated rRsbW has resulted in roughly two peaks, whereas, other GdnCl-exposed samples primarily yielded a single peak (Fig 6D). The elution volumes of 0, 0.5, 2, and 3 M GdnCl-equilibrated rRsbW were 80.5, 83.3, 76.23, and 75.25 ml, respectively. Conversely, two peaks of 1 M GdnCl-exposed rRsbW correspond to 82.38 and 75.25 ml, respectively. A‘shoulder’ peak with the retention volume of ~75 ml was also noticed in the presence of 0.5 M GdnCl. Such data have strongly supported our hypothesis that there is the formation of two stable rRsbW intermediates in the presence of GdnCl. The above chromatographic data also have provided vital clues about their shapes. The rRsbW intermediate formed at 0.5–1 M GdnCl has a relatively compressed shape compared to native rRsbW as it was eluted at a greater elution volume. In contrast, the shape of intermediate generated at ~2.3 M GdnCl is comparatively larger than that of native rRsbW as the former was eluted prior to the elution of latter.

To test whether the GdnCl-made intermediatesare biologically active, astandard serine kinase assay at 0–2.5 M GdnCl was carried out as demonstrated above. Fig 6Ereveals the production of rRsbW-catalyzed phosphorylated rRsbV mostly at 0–1 M GdnCl. The plot of theamount of phosphorylated rRsbV versus GdnCl concentrations show that there is about 10% loss of kinase activity at 0.5 M GdnCl (Fig 6F). The activity was lost higher than 40% when the GdnCl concentration was raised to 1 M. At ~1.5–2.5 M GdnCl, rRsbW retained very little or no kinase activity. As there is atrivial alteration of rRsbVstructure during assay time in the presence of 0–5 M GdnCl (S3 Fig), therRsbW intermediate formed at the higher GdnCl concentration is mostly inactive, whereas,another intermediate is somewhat functional at ~0.5–1 M GdnCl.

## Discussion

Multi-domain proteins usually use their different domains to perform different functions [41, 42]. Conversely, single-domain proteins most times use their different/overlapping regions or motifs to fulfilldifferent objectives [31, 43]. RsbW-like bacterial regulators may have different domains/regions for binding SigB, interacting with RsbV, carrying out phosphorylation, holding ATP, and for forming dimers [1–3]. Our limited proteolysis data with rRsbW (Fig 2) have ruled out the presence of multiple domains in the RsbW proteins and convincingly show that these multifunctional regulators are single domain-proteins. Three proteolytic enzymes used in the present study can together recognize the peptide bonds formed by ten different amino acid residues of rRsbW (Fig 2A), further indicating the absence of exposed flexible region in the RsbW proteins. Additionally, our proteolysis data partly verified the single-domain structure of SpoIIAB [25], a distant relative of RsbW [1].

**Fig 7:**
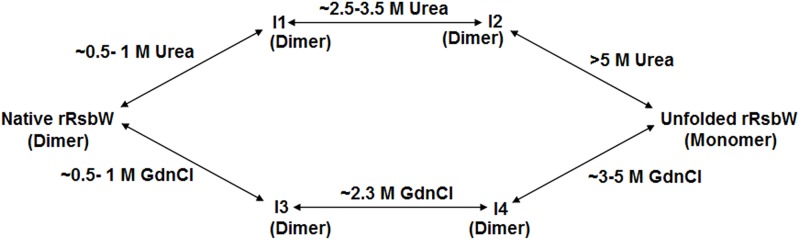
A schematic representation of the urea- and GdnCl-induced unfolding of rRsbW. Intermediates are indicated by I1 to I4.

Our investigations also demonstrated that both the urea- and GdnCl-induced unfolding of rRsbW proceeded via the generation of at least two stable intermediates (Fig 7). Unfolding pathway of rRsbW in the presence of either chemical denaturant was reversible despite the formation of intermediates (S2 and S4 Figs). All of the rRsbW intermediates (designated I1 to I4) roughly exist as the dimers in the aqueous solution (Figs 4 and 6). Other properties of intermediates are, however, quite different from each other. Of the intermediates, only I3 seems to carry a molten globule-like structure (Fig 6). The I3 molecules, generated at relatively small GdnCl concentrations, are partially active and have a shape, which is smaller than that of native rRsbW (Fig 6). Conversely, the other GdnCl-made intermediate (I4) lacks enzymatic activity and significantly differs from both I3 and native rRsbW at the structural and shape levels (Fig 6). The rRsbW intermediate I1, which was produced at the comparatively low urea concentrations, also has a smaller dimension as does I3 (Fig 4). However, I1, unlike I3, is not a molten-globule [38] and has lost almost no kinase activity. The other urea-made intermediate (I2) also retained most of the enzymatic activity and carried a shape that was nearly similar to that of native rRsbW (Fig 4). However, secondary structure, tertiary structure, and the hydrophobic surface area of I2 are different from those of either I1 or native rRsbW (Figs 3 and 4).

The mechanisms of actions of different protein unfolding agents (e.g. urea, GdnCl, heat, etc.) have not been clearly demonstrated [44, 45]. Apparently, these denaturants unfold proteins by disturbing their non-covalent interactions/bonds. However, different denaturants may unfold a protein by a dissimilar mechanism [31, 39, 46–48]. Of the denaturants used in the present study, GdnCl ionizes in the aqueous solution and produces Gdn^+^ and Cl^-^ ions. The Gdn^+^ ions those were generated at ~0.5–1 M and ~2.3 M GdnCl solution perhaps differently neutralized the negative charges on the rRsbW surface. It in turn differently affected the ionic interactions in rRsbW and led to the generation of two intermediates those werenot only structurally and functionally different from each other but also from the urea-made rRsbW intermediates. Currently, the determinants responsible for the formation of the urea-generated rRsbW intermediates are not clearly known.

Different regions/domains of various proteins have shown different sensitivity to the denaturing agents [49–51]. An examination of our data reveals that upon increasing [urea] from 4 M to 7 M, rRsbW dimer content falls from ~95% to ~35% (Fig 4D), while kinase activity drops from ~80% to ~5% (Fig 4F). Similarly, for [GdnCl] from 0.5 M to 2.5 M, dimer content reduces from ~75% to ~60% (Fig 6B) and kinase activity decreases from ~90% to ~15% (Fig 6F). Therefore, for both denaturants, the kinase activity is more sensitive to the increasing concentration of denaturant. The dimerization site and the ATP binding site/catalytic site in a geobacillus anti-sigma factor SpoIIAB are located at the extreme N-terminal end and nearly at the middle of this molecule, respectively [52]. An alignment of RsbW sequence with that of homologous SpoIIAB suggests that the dimerization region and the ATP binding site/catalytic site in the former protein are possibly composed of amino acid residues ~6–35 and ~46–142, respectively (S5 Fig). The data together suggest a preferential beginning of unfolding from the catalytic center/ATP binding site of rRsbW. In other words, the N-terminal end of rRsbW that may be responsible for its dimerization is relatively less sensitive. The determinants responsible for greater stability or the biological roles of the dimeric region of RsbW proteins are not clearly known.

## Conclusions

The current study for the first time has provided new clues about the folding-unfolding mechanism, and the domain structure of *S*. *aureus* RsbW, ananti-sigma factor. rRsbW,a recombinant RsbW, harbors one domain and unfolds via the generation of two stable intermediates in the presence of urea or GdnCl. All of the intermediates exist as the dimers in the aqueous solution and possess distinct structure. The intermediates formed at low denaturant concentrations have a compressed shape in comparison with that of native rRsbW. Conversely, the intermediate produced at higher GdnCl concentration acquires comparatively a larger shape. On the other hand, the intermediate made by higher urea concentrations shows very little alteration of shape. Of the intermediates, the intermediate generated at low GdnCl concentrations owns a molten globule-like structure. Each chemical-induced unfolding reaction was also reversible in nature. Additionally,the putative ATP binding site/catalytic site of rRsbW looks more denaturant sensitive than the remainder of this molecule. The unfolding data may be useful to screen the new antibacterial compounds in the future.

## Supporting information

S1 Fig Urea-induced unfolding of rRsbW. Far-UV CD (A), ANS fluorescence (B), and the intrinsic Tyr fluorescence (C) spectra of rRsbW in the presence of indicated concentrations of urea. (D) Far-UV CD spectra of rRsbV at 0–7 M urea. Protein was treated with urea for 20 min at room temperature prior to recording spectra.
(TIF)

S2 Fig Refolding of the urea-treated rRsbW. Far-UV CD (A), and intrinsic Tyr fluorescence (B) spectra of unfolded, refolded, and native rRsbW. (C) Kinase activity of refolded rRsbW.
(TIF)

S3 Fig GdnCl-induced unfolding of rRsbW. Far-UV CD (A), ANS fluorescence (B), and the intrinsic Tyr fluorescence (C) spectra of rRsbW in the presence of indicated concentrations of GdnCl. (D) Far-UV CD spectra of rRsbV at 0, 0.5 and 5 M GdnCl. Protein was exposed to GdnCl for 20 min at room temperature before recording spectra.
(TIF)

S4 Fig Refolding of the GdnCl-treated rRsbW. Far-UV CD (A), and intrinsic Tyr fluorescence (B) spectra of unfolded, refolded, and native rRsbW. (C) Kinase activity of refolded rRsbW.
(TIF)

S5 Fig Alignment of the *S*. *aureus* RsbW with *G*. *stearothermophilus*SpoIIAB. Sequences of proteins were aligned by ClustalW program. The dimerization site and the ATP binding site [NG1G2G3]/catalytic site of *G*. *stearothermophilus*SpoIIAB are shown as stated [52].
(TIF)
