# Supplementary figures and images for: A staphylococcal anti-sigma factor possesses a single-domain, carries different denaturant-sensitive regions and unfolds via two intermediates
Source: PLoS One. 2018 Apr 5;13(4):e0195416. doi: 10.1371/journal.pone.0195416 (PMC5886543; doi:10.1371/journal.pone.0195416)

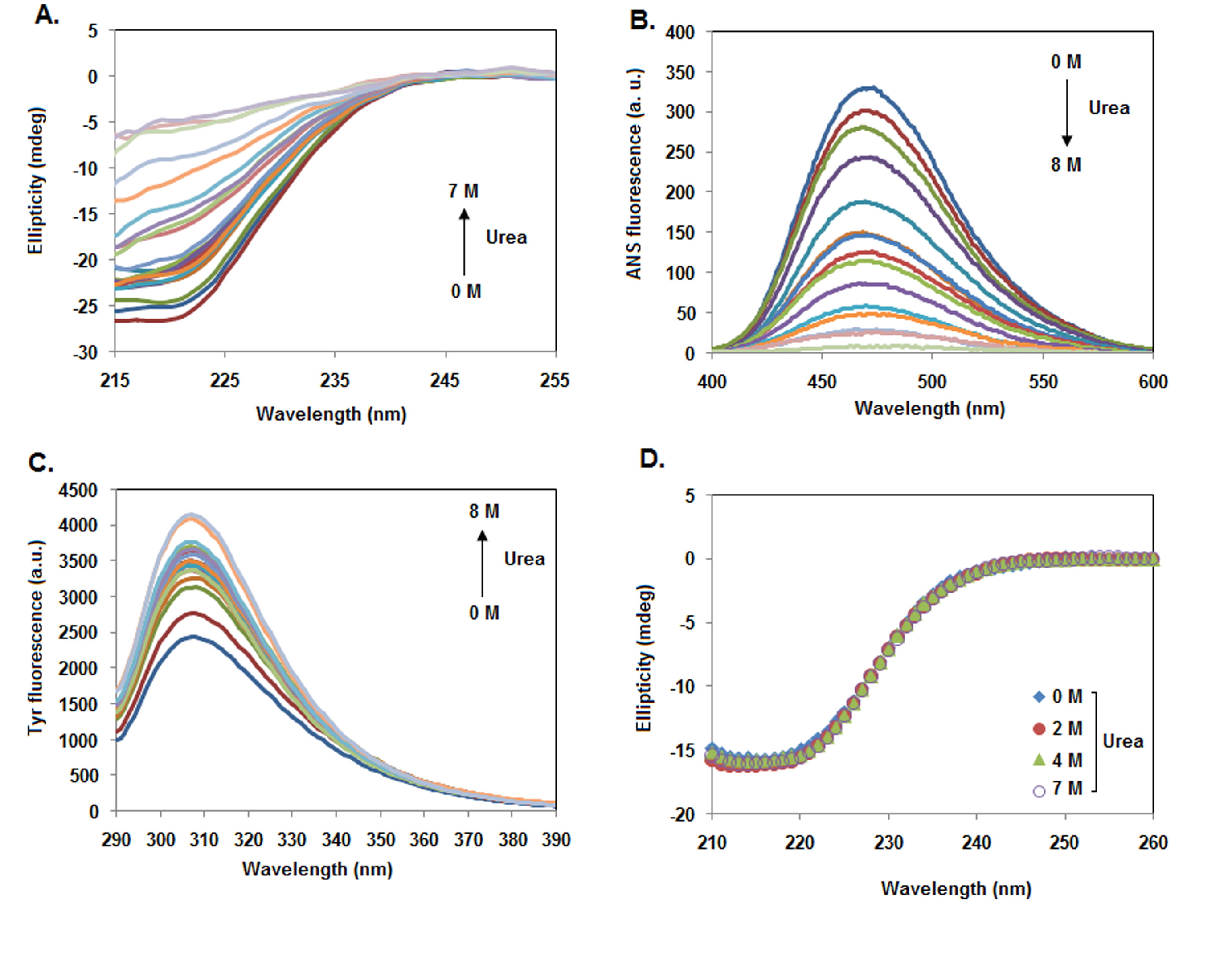

Supplement: S1 Fig — Far-UV CD (A), ANS fluorescence (B), and the intrinsic Tyr fluorescence (C) spectra of rRsbW in the presence of indicated concentrations of urea. (D) Far-UV CD spectra of rRsbV at 0–7 M urea. Protein was treated with urea for 20 min at room temperature prior to recording spectra. (TIF) [file pone.0195416.s001.tif]

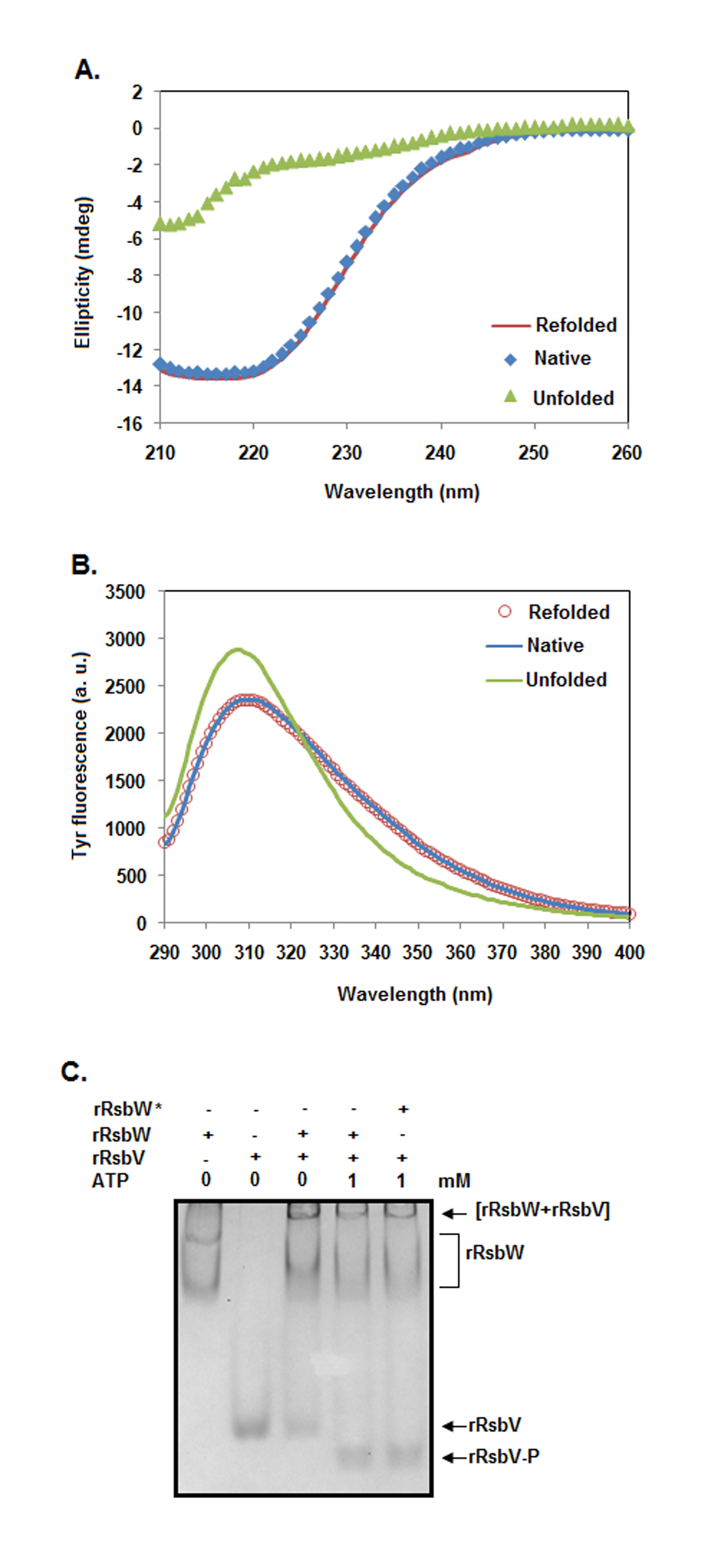

Supplement: S2 Fig — Far-UV CD (A), and intrinsic Tyr fluorescence (B) spectra of unfolded, refolded, and native rRsbW. (C) Kinase activity of refolded rRsbW. (TIF) [file pone.0195416.s002.tif]

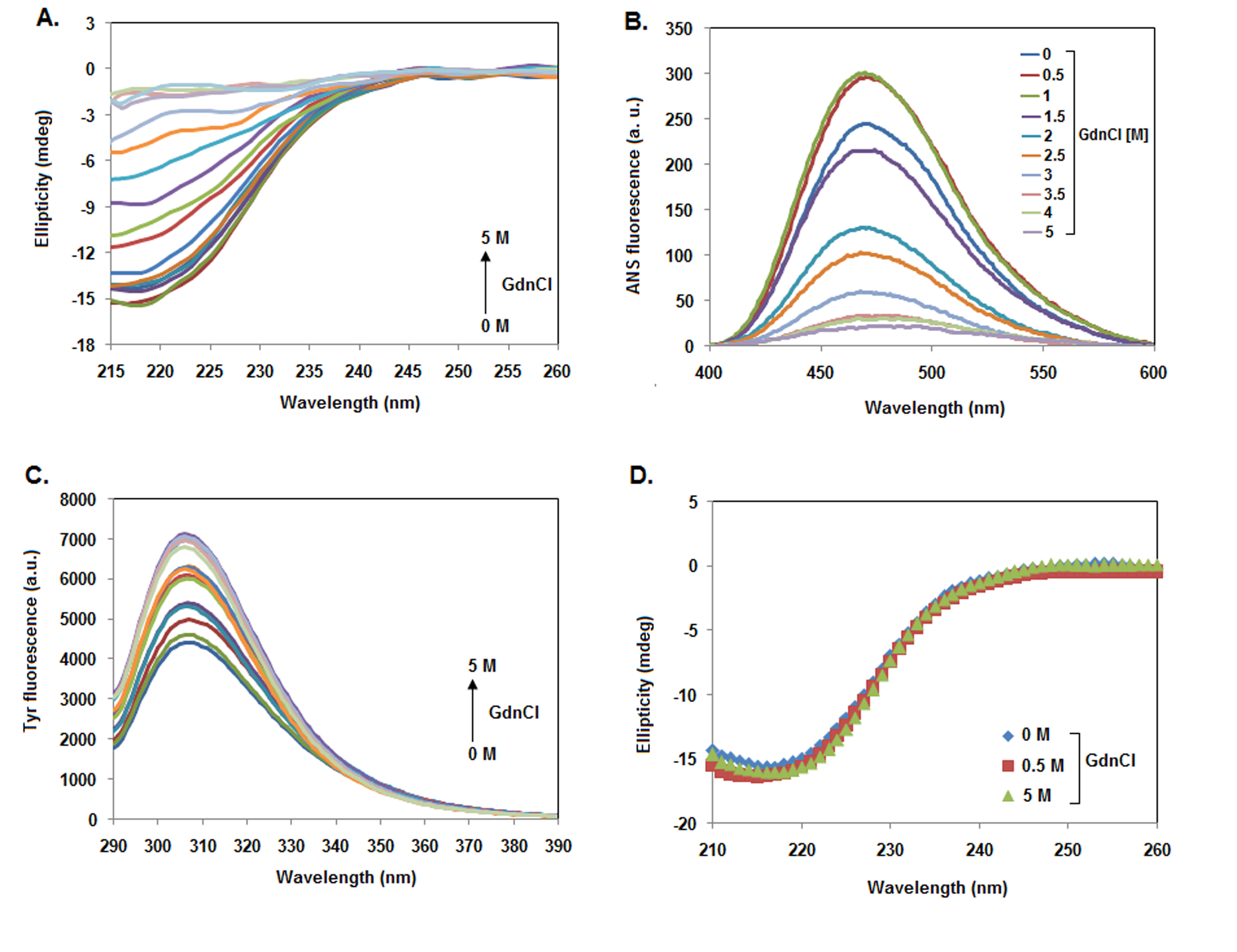

Supplement: S3 Fig — Far-UV CD (A), ANS fluorescence (B), and the intrinsic Tyr fluorescence (C) spectra of rRsbW in the presence of indicated concentrations of GdnCl. (D) Far-UV CD spectra of rRsbV at 0, 0.5 and 5 M GdnCl. Protein was exposed to GdnCl for 20 min at room temperature before recording spectra. (TIF) [file pone.0195416.s003.tif]

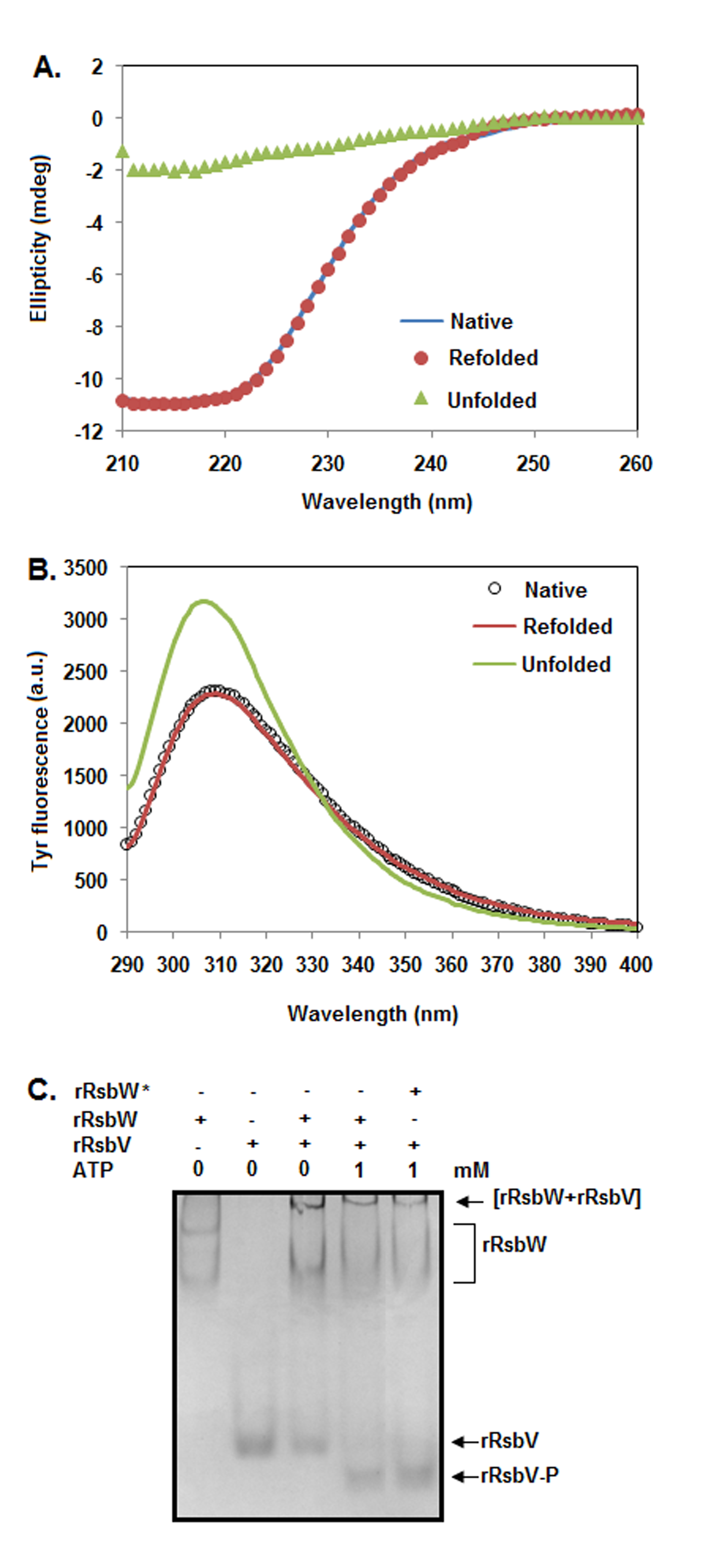

Supplement: S4 Fig — Far-UV CD (A), and intrinsic Tyr fluorescence (B) spectra of unfolded, refolded, and native rRsbW. (C) Kinase activity of refolded rRsbW. (TIF) [file pone.0195416.s004.tif]

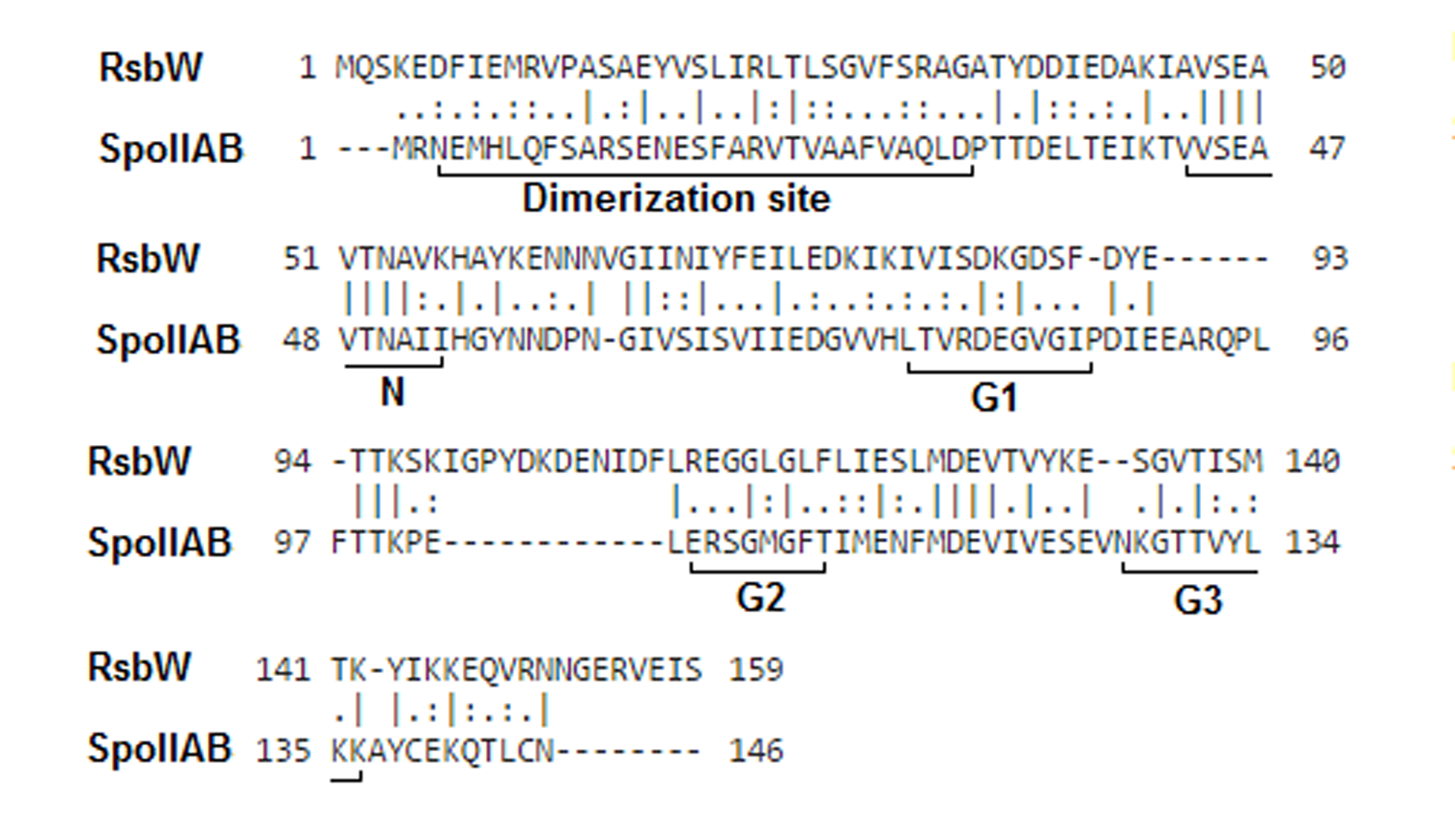

Supplement: S5 Fig — Sequences of proteins were aligned by ClustalW program. The dimerization site and the ATP binding site [NG1G2G3]/catalytic site of G. stearothermophilusSpoIIAB are shown as stated [52]. (TIF) [file pone.0195416.s005.tif]
